# Self-Reducible Painless and Recurrent Prosthetic Hip Dislocation: A Case Study

**DOI:** 10.7759/cureus.73706

**Published:** 2024-11-14

**Authors:** Frances Akwuole, Mitchell Pfennig, Whisper Grayson, Nicholas Brown

**Affiliations:** 1 Orthopaedic Surgery, Loyola University Chicago Stritch School of Medicine, Maywood, USA; 2 Orthopaedic Surgery, Loyola University Medical Center, Maywood, USA

**Keywords:** closed reduction of hip, hip heterotopic ossification, spino-pelvic alignment, spinopelvic alignment, total hip arthroplasty complication, total hip arthroplasty dislocation

## Abstract

Prosthetic hip dislocations following total hip arthroplasty can significantly impact patient quality of life and functional capabilities. Early dislocations typically occur within the first three months post-surgery, while delayed dislocations arise after three months. Notably, patients may experience implant instability and dislocation for years, even decades, after the initial procedure due to a variety of underlying issues. A comprehensive evaluation including patient history, physical examination, and imaging studies is essential for diagnosing delayed dislocations.

Reducing prosthetic hip dislocations can be particularly challenging, often necessitating the cooperation of multiple healthcare professionals to perform a series of reduction maneuvers. For this reason, it is surprising when an individual is able to self-reduce a dislocated prosthetic hip. Documenting this instance of self-reduction can foster dialogue among orthopedic surgeons and healthcare providers, ultimately enhancing the management strategies for similar cases in the future.

In this study, the case of a 73-year-old male with a six-month history of painless, recurrent prosthetic hip dislocations with self-reduction is detailed. Self-reduction was performed via maneuvers including right lower extremity extension and external rotation. To corroborate the patient’s story, multiple X-rays were obtained. These images demonstrated an initially stable right hip prosthesis, followed by evidence of a dislocated femoral implant, and concluded with a reduced hip after self-reduction.

Self-reduction of a prosthetic hip dislocation by a patient is unusual, therefore presenting a unique case. The primary purpose of this case report is to describe this case of self-reduction, increase awareness of this instance, and highlight the importance of obtaining serial imaging to thoroughly identify a potential dislocation.

## Introduction

Total hip arthroplasty (THA) stands as a transformative surgery that enhances mobility and restores function in patients with hip arthritis [1]. Implant instability and dislocation are common complications of THA, often necessitating revision arthroplasty [2,3]. Risk factors for dislocation include non-compliance with postoperative restrictions, component malposition or failure, lumbar immobility, and inadequate restoration of soft tissue tension [4]. In cases of prosthetic hip dislocation, reduction in the emergency department under conscious sedation is usually attempted first, with irreducible joints requiring reduction in the operating room under general anesthesia. These reduction maneuvers are often intricate, requiring multiple sets of hands for successful reduction and therefore difficult to perform by oneself [5].

Recurrent hip dislocations and atraumatic prosthetic dislocations are not uncommon, with numerous cases reported in the literature [6,7]. Adrados et al., for example, detailed two cases of women who experienced late, atraumatic, posterior total hip arthroplasty dislocations from yoga exercises [8]. Despite hip dislocation being a common complication following arthroplasty, there is little in the literature on patients who repeatedly reduced their atraumatic prosthetic hip dislocation without the assistance of health care professionals. This report presents the case of a patient who performed serial reductions on an atraumatic prosthetic hip dislocation, highlighting the necessity of serial imaging to ensure accurate diagnosis.

## Case presentation

A 73-year-old male with a surgical history of left total hip arthroplasty in 2019 and right total hip arthroplasty in 2000 for primary osteoarthritis (posterior approach) was admitted to acute inpatient rehabilitation, February 2024, one day after undergoing a left total knee arthroplasty (TKA) for treatment of primary osteoarthritis.

During his admission and rehabilitation for his left total knee arthroplasty, the patient reported a six-month history of intermittent atraumatic and painless displacement of his right hip. According to the patient, when his hip dislocated, he was unable to fully extend his right lower extremity and was able to reduce the hip by himself through maneuvers such as right lower extremity extension and external rotation.

**Figure 1:**
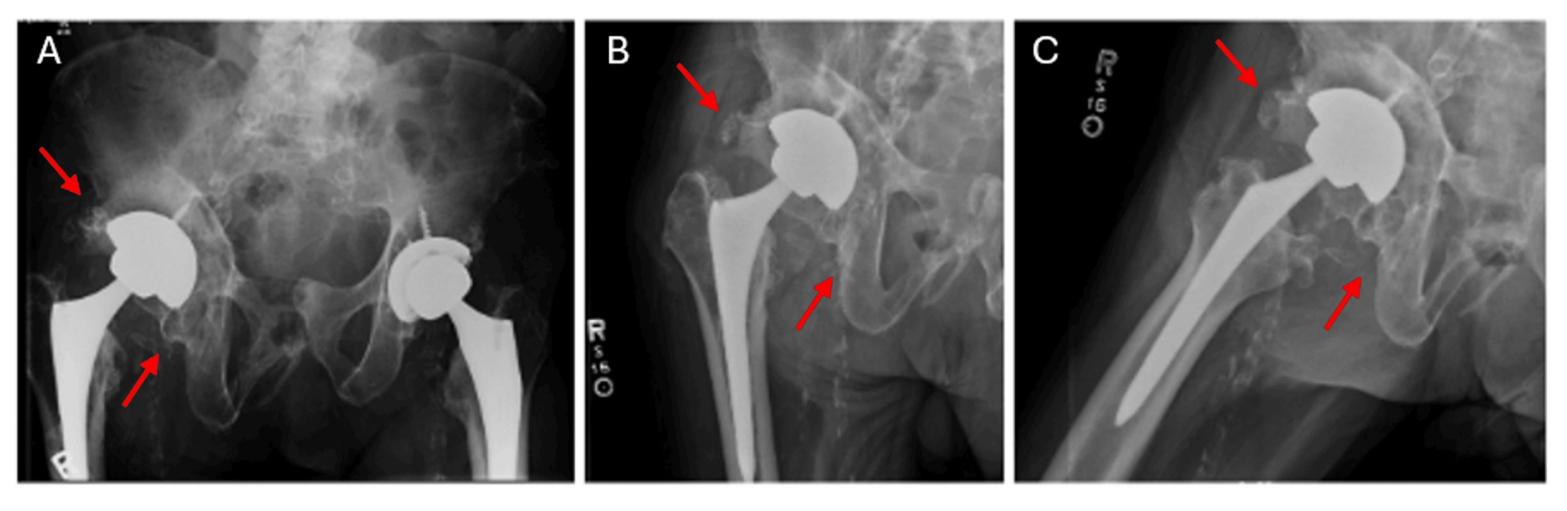
Initial Plain Films at Time of Rehabilitation Admission A: Anteroposterior view of the pelvis demonstrating a stable hip implant without evidence of fracture, dislocation, or loosening of the acetabular or femoral component. The femoral head is in anatomic axis. There is presence of heterotopic ossification at the superolateral and inferomedial aspect of the acetabulum. B: Anteroposterior view of the right hip demonstrating a stable hip implant without evidence of fracture, dislocation, or loosening of the acetabular or femoral component. The femoral head is in anatomic axis. There is presence of heterotopic ossification at the superolateral and inferomedial aspect of the acetabulum. C: Cross table lateral of the right hip demonstrating a stable hip implant without evidence of fracture, dislocation, or loosening of the acetabular or femoral component. The femoral head is in anatomic axis. There is presence of heterotopic ossification at the superolateral and inferomedial aspect of the acetabulum.

On exam, a posterior pelvic tilt was noticeable, as well as a limb length discrepancy, with his right lower extremity being shorter than the left. Upon repeat exams, a series of clicks and clunks could be palpably felt when flexing and extending the hip. The first set of plain films were obtained and demonstrated a stable prosthetic hip implant without obvious dislocation or fracture, and a large heterotopic ossification (HO) lesion surrounding the acetabulum (Figure 1A-1C). At this time, however, the patient stated that he felt his hip was in place during the imaging.

**Figure 2:**
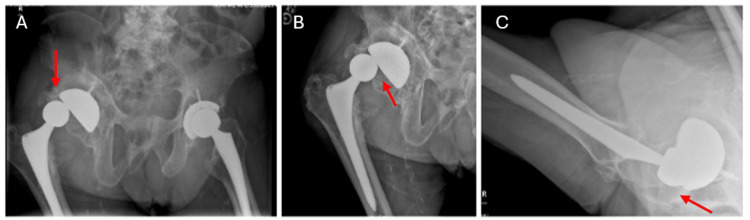
Secondary Set of Plain Films A: Anteroposterior views of the pelvis demonstrating superolateral displacement of the right femoral head from the acetabular component without evidence of fracture or loosening of the acetabular or femoral component. There is presence of heterotopic ossification at the superolateral and inferomedial aspect of the acetabulum. B: Anteroposterior views of the right hip demonstrating superolateral displacement of the right femoral head from the acetabular component without evidence of fracture or loosening of the acetabular or femoral component. There is presence of heterotopic ossification at the superolateral and inferomedial aspect of the acetabulum. C: Cross table lateral of the right hip demonstrating posterior displacement of the right femoral head from the acetabular component without evidence of fracture or loosening of the acetabular or femoral component. There is presence of heterotopic ossification at the superolateral and inferomedial aspect of the acetabulum.

**Figure 3:**
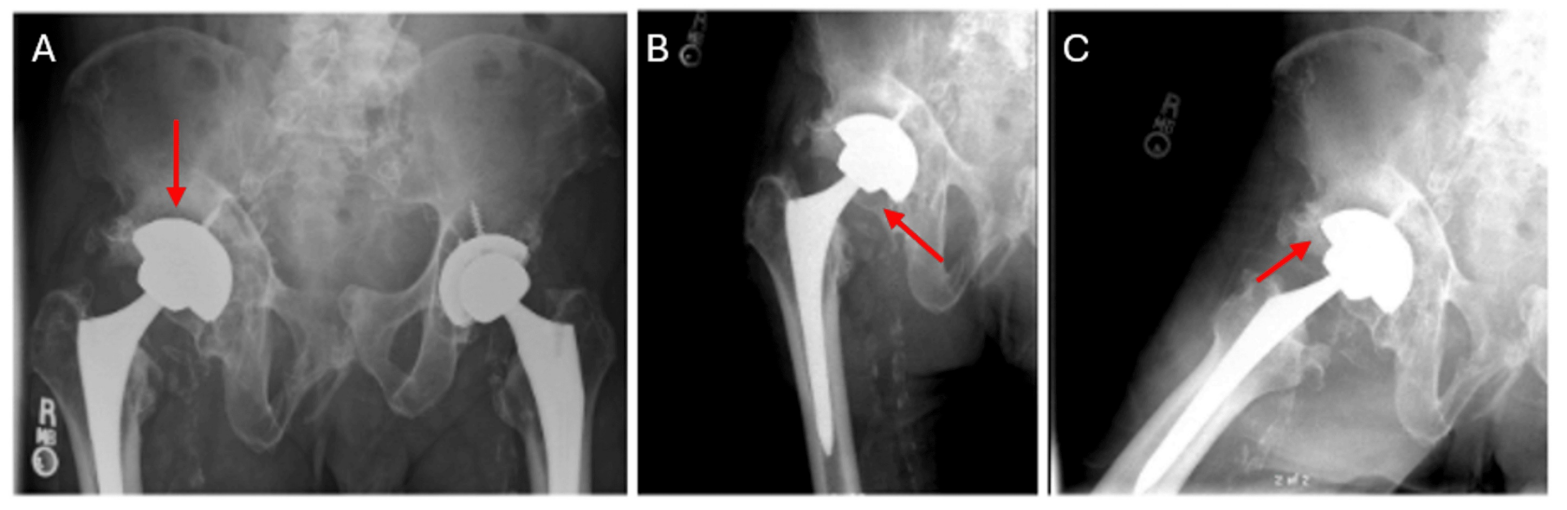
Post-Self-Reduction Plain Films A: Anteroposterior view of the pelvis demonstrating a reduced and stable hip implant without evidence of fracture, dislocation, or loosening of the acetabular or femoral component. The femoral head is relocated in the hip joint and there is continued presence of heterotopic ossification. B: Anteroposterior view of the right hip demonstrating a reduced and stable hip implant without evidence of fracture, dislocation, or loosening of the acetabular or femoral component. The femoral head is relocated in the hip joint and there is continued presence of heterotopic ossification. C: Cross table lateral of the right hip demonstrating a reduced and stable hip implant without evidence of fracture, dislocation, or loosening of the acetabular or femoral component. The femoral head is relocated in the hip joint and there is continued presence of heterotopic ossification.

**Figure 4:**
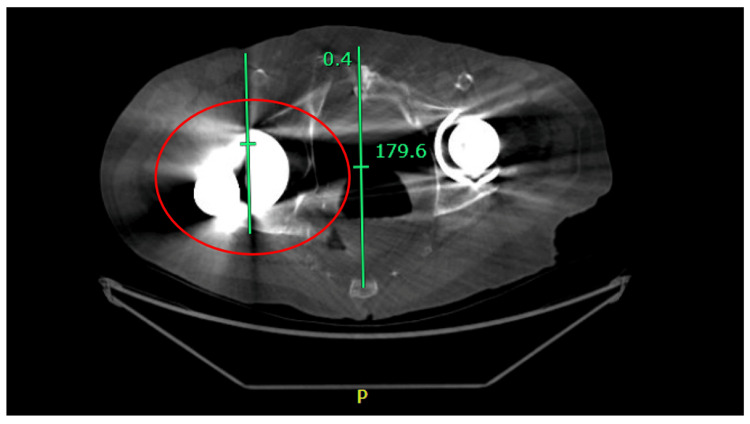
Computed Tomography Scan of the Pelvis Above is a choice axial CT image of the dislocated hip demonstrating lateral translocation of the right femoral head with the acetabular cup remaining stable in neutral positioning.

The decision was made to order another set of plain films to be taken while he felt that his hip was again displaced. These new images demonstrated a posterior and superolateral dislocation of the right femoral head from the acetabular component, without any signs of acute fracture (Figure 2A-2C). Post-self-reduction films showed evidence of a reduced right prosthetic implant (Figure 3A-3C). Computed Tomography (CT) was obtained at a different time point during one of the reported dislocations, which also demonstrated a posterior and superolateral dislocation (Figure 4). Based on these findings, the decision was made to proceed with a revisional total hip arthroplasty, however, the patient declined reoperation.

## Discussion

To our knowledge, this is the only documented report on self-reduction of a prosthetic hip dislocation. Initially, the diagnosis for this patient was a snapping hip syndrome with iliopsoas/iliotibial symptoms of audible pops and clicking sensations present upon examination [9]. While snapping hip syndrome aligned with the patient’s history and presentation, the limb length discrepancy could not be explained. Thus, obtaining a repeat set of X-rays when the patient felt that the hip was out of place was crucial in the identification of the dislocation (Figure 2A-2C). Subsequent X-rays while the patient felt that the hip was reduced confirmed that he indeed was able to self-reduce the hip without pain or assistance (Figure 3A-3C).

Atraumatic dislocations following total hip arthroplasty are often multifactorial [10]. Our patient’s dislocations may have been due to leg length discrepancy causing inadequate restoration of soft tissue, a relatively neutral cup position, and stiffening of his spine due to degenerative changes. Additionally, the dislocated femoral head may have been somewhat contained by the heterotopic ossification keeping it in a reasonable enough position to allow for self-reduction.

These potential causes align with the current literature, investigating common contributors to hip dislocation. When evaluating the spinopelvic biomechanical relationship, hip dislocations may frequently be caused by spinal pathology, with recent studies demonstrating increased risk of hip dislocation and revision in patients with a prior history of lumbar spinal or lumbosacral fusions [11-13]. Risk factors for adverse spinopelvic mobility include but are not limited to stiff lumbar spine, large posterior pelvic tilt, and severe sagittal spinal deformity [14]. Though our patient did not have adequate imaging for radiographic measurements of the spine and pelvis, a posterior pelvic tilt was detected on physical exam. In addition, our patient did not have any prior spinal surgeries or fusions but does have degenerative changes of the spine and posttraumatic changes of the pelvis due to pelvic fractures sustained in 1982.

Acetabular cup positioning is another important factor, with studies showing a slightly anteverted cup to be protective against dislocation [15]. Conversely, excessive anteversion has been shown to be associated with increased dislocation risk [16]. While this can serve as a perioperative guide, the optimal cup position is often patient-specific, depending upon femoral version, pelvic position, and spinopelvic mobility [17-19]. Thus, while no formal diagnosis was able to be achieved, this patient’s neutral cup position in combination with presumed minimal spinal pelvic mobility was likely a contributor to the dislocations. 

Despite hip dislocations being a not-uncommon occurrence, self-reduction is less frequently commented upon. Though instances of self-reductions for shoulder dislocations have been widely recognized, there are sparse reports of self-reductions in the lower extremity literature. For example, Dudkiewicz et al. highlighted the success of teaching patients a modified Milch technique for reducing their own shoulders without the need for healthcare provider assistance [20]. However, to our knowledge, this is the first report detailing a case of self-reduction following recurrent periprosthetic hip dislocations. It is hoped this case will contribute to the paucity of literature on self-reduction of hip dislocations and highlight the importance of serial imaging when making a diagnosis.

## Conclusions

In this report, we presented the case of a patient with recurrent, atraumatic prosthetic hip dislocations resolved with self-reductions. Completing a detailed physical exam and obtaining serial imaging was essential in confirming the diagnosis. Patients who indicate a concern about the stability of their hip prosthesis should be assessed thoroughly. Obtaining plain films at just one point in time may not be sufficient enough to appropriately discover a pathology. It is important to make orthopaedic surgeons aware of the possibility that a patient can self-reduce a prosthetic hip dislocation. This recognition can help prevent false convictions of a stable implant in instances where a revisional arthroplasty would instead be necessitated. This report presents a unique case, worthy of discussion, to further understand potential underlying pathological implications that may lead to recurrent dislocations and self-reductions.
